# Anomalous Incidence of Fatal Musculoskeletal Injury in North American 2-Year-Old Thoroughbred Racehorses in the Year 2020

**DOI:** 10.3390/ani13162572

**Published:** 2023-08-09

**Authors:** Euan D. Bennet, Tim D. H. Parkin

**Affiliations:** 1 School of Biodiversity, One Health, and Veterinary Medicine, Garscube Campus, University of Glasgow, Glasgow G61 1QH, UK; 2 Bristol Veterinary School, Langford Campus, University of Bristol, Bristol BS40 5DU, UK; tim.parkin@bristol.ac.uk

**Keywords:** Thoroughbred racehorse, catastrophic injury, fatality, musculoskeletal

## Abstract

**Simple Summary:**

In the early stages of the global COVID-19 pandemic, many countries implemented strong anti-infection mitigations. In the United States and Canada, these measures affected the training regimens of Thoroughbred racehorses—pandemic mitigations meant that ‘normal’ training schedules could not be followed. These circumstances led to a natural experiment: many 2-year-old Thoroughbreds, who would normally have begun intensive preparation for starting their racing careers in early 2020, had the beginning of their training delayed. Meanwhile, horses aged 3 years and older also experienced an interruption to their normal or expected training programmes. On full analysis of the 2020 annual statistics on behalf of The Jockey Club, an increase in the number of fatal musculoskeletal injuries among 2-year-old horses compared to previous years was observed. No similar increase was seen among horses aged 3 years and older in 2020. In 2020, 2-year-olds were the age group with the highest risk of fatal injury. In 2021 and 2022, the incidence of fatalities in 2-year-old horses returned to the previous trend. In all previous (to 2020) and the two subsequent years, the risk of fatal musculoskeletal injury has been lowest in 2-year-old horses, compared with 3- and 4+-year-olds. This result emphasises the importance of racehorses following a suitable training schedule, especially for racehorses at the beginning of their career.

**Abstract:**

Racehorse training and racing schedules in many parts of the United States and Canada were interrupted or otherwise reduced during the first three to six months of 2020. This was an indirect consequence of mitigations to prevent the spread of the pandemic virus COVID-19. Data from the Equine Injury Database, a census-level survey of all race starts made in the USA and Canada, were used to analyse the incidence of fatalities in 2009–2022 among three age cohorts of racehorses within each year. There was a statistically significant increase in the incidence of musculoskeletal fatalities among 2-year-old Thoroughbreds in 2020, compared to the period 2009–2019. In 2021 and 2022, the training schedules of 2-year-old horses returned to pre-2020 levels, as did the incidence of fatalities. The delayed start to training for 2-year-old horses was associated with an increase in risk of fatal musculoskeletal injury for those horses during 2020, but the risk for the same horses in 2021—when they were 3 years old—was not significantly different to the risk for 3-year-olds in any other year. The increased risk of fatal musculoskeletal injury in 2020 was only found among horses that were 2 years old in 2020—horses aged 3 years or more in 2020 were not at increased risk.

## 1. Introduction

Thoroughbred racehorses require carefully managed training programmes in order to maximise well-being and competitiveness. Horses must be suitably prepared for the physical stress of racing if they are to be successful. Ideally, trained horses will be ready to both be a challenger to win any given race, while also being physically prepared enough to minimise the risk of injury. The literature has identified risk factors for race-associated deleterious outcomes, including serious or fatal musculoskeletal injury [1,2,3,4,5,6,7,8,9,10,11,12,13,14,15,16,17,18,19] and sudden death [20,21,22,23,24]. Similar outcomes have been reported in training [25,26]. An important factor that has been identified relating to appropriate training schedules is bone adaptation. Careful management of racehorse workloads is required to avoid bone microdamage accumulation [27,28,29,30].

Racing and, more generally, the use of horses in sports have recently been subject to increased public scrutiny, with discussions around the social licence to operate (SLO) of equine sports reaching increased prominence compared to previous years [31]. To protect the SLO, sports governing bodies must not only act, but also be transparent and visible in their actions. As such, in the event of, say, a high-profile fatal injury to a racehorse, or a cluster of several fatal injuries within a short time period, governing bodies are under pressure to respond proactively.

The global COVID-19 pandemic that began in 2020 has caused significant disruption across society in many countries. Many aspects of society were affected by the direct effects of the pandemic virus (i.e., widespread sickness and death) and were also indirectly affected by public health mitigations introduced to limit the direct effects. In many countries, mitigations were introduced that temporarily required businesses to reduce or cease operation. In the USA and Canada, many racetracks were affected and, as a result, the racehorses managed at those tracks experienced a reduction in both their training and racing schedules for a period of up to three months during 2020.

The outcome of this situation was that a natural experiment occurred. In early 2020, a cohort of 2-year-old Thoroughbreds who were preparing to begin their racing careers had both their intensive training and the start of their careers delayed. Older cohorts of horses aged 3 years and above experienced an interruption to their usual training programmes.

This retrospective descriptive cohort study investigated whether training schedules of Thoroughbred racehorses were disrupted in 2020. Potential associations between the disruption to training and fatal musculoskeletal injury were examined. The data used were from the Equine Injury Database (EID)—a census-level report of Thoroughbred flat racing in the USA and Canada collected by The Jockey Club. The incidence of racehorse fatalities—caused by fatal musculoskeletal injuries or otherwise—has been on a sustained downward trend since 2019. In 2022, the incidence was 38% lower than in 2009 [32].

It was hypothesised that a delayed start to training and racing for 2-year-old horses could be associated with an increase in the incidence of musculoskeletal fatalities. It was also hypothesised that an interruption to the usual training schedule of horses aged 3 years and older could be associated with an increase in the incidence of musculoskeletal fatalities among that cohort.

## 2. Materials and Methods

The full EID was available for this study. The EID contained records of every Thoroughbred flat race held in the USA and Canada between 1 January 2009 and 31 December 2022, totalling 4,466,870 horse starts made by 299,683 individual horses. Additionally, 8,532,608 training records were available to be reconciled with the race data, also recorded by The Jockey Club as a supplemental part of the ‘main’ EID. The study cohort comprised every ‘trackwork start’ between 1 January 2015 and 31 December 2022—where one trackwork start was one horse starting in one race or recorded training workout. The case definition was any fatality recorded within three days of racing, and which was attributed to musculoskeletal injury.

The final study cohort was 6,341,511 trackwork starts made by 163,878 individual horses. This included 2,267,032 race starts and 4,074,479 recorded workouts made by the same horses. Chi-squared tests were used to test associations between the incidence of fatality in each year, and for continuous variables, the Mann–Whitney test was used to compare between years.

## 3. Results

### 3.1. Musculoskeletal Fatalities

**Figure 1:**
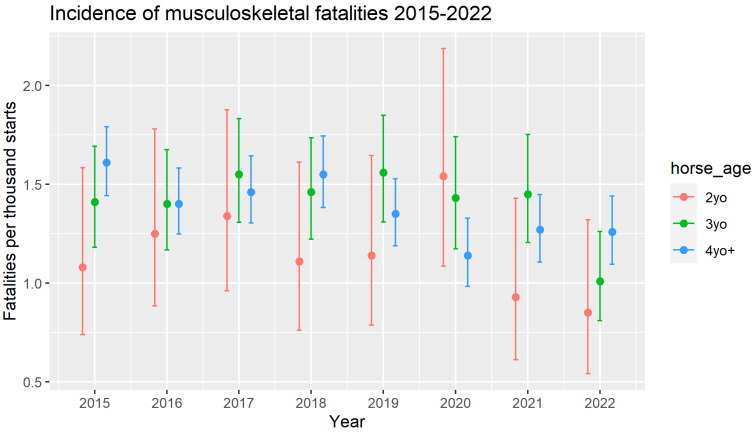
The incidence of fatal musculoskeletal injuries recorded in the Equine Injury Database, 2015–2022. The incidence per thousand race starts is shown for each age group of horse: 2 years old, 3 years old, and 4 years and older.

Figure 1 and Table 1 show the incidence of fatal musculoskeletal injury among each of three age groups of horses: 2-year-olds, 3-year-olds, and 4-year-olds and older. In every year except for 2020, the incidence of fatal musculoskeletal injury was lowest in the 2-year-old group. There was an increase in incidence of musculoskeletal fatalities only amongst 2-year-olds in 2020, which resulted in that age group having the highest incidence for the first and only time during the study period. This change was not statistically significant compared to the previous five-year average, according to the chi-squared test (*p* = 0.4). There was no commensurate increase among horses aged three years and older in 2020. Furthermore, there was no ongoing increase in risk in 2021 or 2022 among horses that were 2 years old in 2020. The trend of 2-year-old horses having the lowest incidence of fatalities was restored in 2021 and continued into 2022. The reduction in incidence of fatalities from 2020 was statistically significant at the 90% confidence level for both 2021 (*p* = 0.09) and 2022 (*p* = 0.05).

### 3.2. Training Schedule

Table 2 shows the median number of training workouts completed prior to a horse’s first race start in each year. It also shows the median days between a horse’s first training workout in a year, and their first race start in that same year. Training schedules in 2020, once started, were not statistically significantly different from those in any other year. The number of training workouts completed, and the time between starting training and the first race start, were consistent across all years for each age group.

### 3.3. Days to First Work and First Race Each Year

Although training schedules—in terms of the number of training workouts and time from the start of training to first race start—were not significantly different in 2020, the start dates of 2-year-old horses entering training and entering racing were both delayed on average. Table 3 shows the median days to first training workout, and to first race start, in each year. In every year, there is a substantial difference between the 2-year-old cohort and the older age groups, because the 2-year-old horses are in their first year of racing while the majority the older age groups are continuing their career from the previous year. In 2020, the median days to the first training workout for 2-year-old horses was, on average, 12 days (range 8–27 days) higher than in any other year in the study period. This difference was statistically significant compared to each other year (*p* < 0.001). Furthermore, in 2020, the median days to first race start for 2-year-old horses was, on average, 9 days (range 7–11 days) higher than in any other year studied, and this difference was also statistically significant (*p* < 0.001).

## 4. Discussion

The incidence of two-year-old racehorse fatalities in the USA and Canada was unusually high in 2020, the only year since 2009 (the first full year of data for which data are available) in which the incidence of fatalities was higher in two-year-olds compared to older ages. In 2021 and 2022, the incidence returned to below the previous ‘normal’ level, and the two lowest incidences of two-year-old fatalities since 2009 were recorded in those successive years. The fatal musculoskeletal injury incidence of three-year-old, and four-years-and-older, horses did not follow the same pattern. For horses that were aged two years in 2020, no commensurate increase in incidence of fatal musculoskeletal injury was found in 2021 (at age of three years) or in 2022 (at age four years). This suggests that the year of 2020 was anomalous only for horses beginning their racing career aged two years. It also suggests that the anomaly of 2020 did not follow the cohort of affected horses in 2021 or 2022. Understanding the potential causes of the apparent cluster of 2-year-old fatalities is crucial for protecting racing’s SLO. Public scrutiny of racehorse fatalities could (perhaps understandably) focus on the apparently anomalous increase in incidence in 2020, regardless of whether or not the increase was statistically significant compared to 2019.

When investigating a potential explanation for this anomalous year, it was found that training intensity was not significantly different in 2020 compared to any other year since 2015. Horses completed the same number of training workouts before entering racing, over almost the same time period. The median days to entering training, and median days to entering racing, were statistically significantly higher among two-year-old horses in 2020 compared to any other year since 2015. On average, two-year-old horses entered training two weeks later in 2020 compared to previous years, and entered racing nine days later in 2020 compared to previous years.

Further investigation is necessary to understand if the anomalous fatal musculoskeletal injury incidence for 2-year-old horses was indeed caused by a delayed start to training. It is possible that while the amount and frequency of trackwork among 2-year-old horses were, on average, not significantly different across the whole of 2020, a more detailed investigation of month by month (or by 3 month blocks, say) may show a more complex pattern. It could be the case, for example, that horses experienced lower-than-expected workloads early in the year, followed by some degree of ‘catch-up’ later, closer to beginning racing. If so, then there is the potential that horses did not have the opportunity to accumulate sufficient work-load cycles to generate appropriate bone adaptation early in the season [29,30].

This delay to entering training is unlikely to fully explain the anomalous year. The median days to entering training was 192 days from the start of the year, i.e., 11th July. It is likely that there are other, unmeasured, factors that were present between the introduction of pandemic mitigations in many states around March 2020, and July 2020 as the median date for two-year-old horses entering training. It should also be noted that several US states never introduced pandemic mitigations during 2020, and some states had much lighter mitigations than others, so a state-level analysis will be necessary to take this into account.

This finding motivates further investigation into the physical mechanisms that underlie fatal musculoskeletal injuries, as well as the specific training patterns of horses that experienced the fatal injuries. That the anomalous incidence of fatal musculoskeletal injury only affected 2-year-old horses suggests that a suitable training schedule at the beginning of their career is an important aspect of racehorse safety. It can be reasonably speculated that bone adaptation driven by training workload cycles is an important factor here [29,30]. Further investigation is required to understand if this finding could, say, point towards an optimal training pattern to minimise risk for both new and experienced racehorses. Careful investigation of complete records including racing and training histories, along with veterinary histories (if available), can lead to evidence-based risk management recommendations that would contribute to welfare-oriented training programs.

## Figures and Tables

**Table 1:** Number of race starts, musculoskeletal fatalities, and fatalities per 1000 starts in each year of the Equine Injury Database from 2015 to 2022, for each of three age groups of horses.

	2-Year-Old Horses			3-Year-Old Horses			4-Year-Old-and-Older Horses		
Year	Starts	Fatalities	per 1000 Starts (95% CI)	Starts	Fatalities	per 1000 Starts (95% CI)	Starts	Fatalities	per 1000 Starts (95% CI)
2015	24,039	26	1.08 (0.74–1.58)	83,513	118	1.41 (1.18–1.69)	201,662	324	1.61 (1.44–1.79)
2016	24,709	31	1.25 (0.88–1.78)	84,362	118	1.40 (1.17–1.67)	195,074	274	1.40 (1.25–1.58)
2017	25,309	34	1.34 (0.96–1.88)	87,207	135	1.55 (1.31–1.83)	194,671	285	1.46 (1.30–1.64)
2018	24,375	27	1.11 (0.76–1.61)	85,829	125	1.46 (1.22–1.73)	184,195	286	1.55 (1.38–1.74)
2019	24,588	28	1.14 (0.79–1.65)	82,895	129	1.56 (1.31–1.85)	181,082	244	1.35 (1.19–1.53)
2020	20,126	31	1.54 (1.09–2.19)	68,600	98	1.43 (1.17–1.74)	147,835	169	1.14 (0.98–1.33)
2021	22,475	21	0.93 (0.61–1.43)	75,036	109	1.45 (1.20–1.75)	166,689	211	1.27 (1.11–1.45)
2022	22,449	19	0.85 (0.54–1.32)	77,130	78	1.01 (0.81–1.26)	163,182	205	1.26 (1.10–1.44)

**Table 2:** Median number (interquartile range) of training workouts completed before the first race start of each year, and median (interquartile) range days between first workout and first race start in each year, 2015–2022, for each of three age groups of horses.

	2-Year-Old Horses		3-Year-Old Horses		4-Year-Old-and-Older Horses	
Year	Median (IQR) Training Workouts before First Race in Year	Median (IQR) Days between First Workout and First Race in Year	Median (IQR) Training Workouts before First Race in Year	Median (IQR) Days between First Workout and First Race in Year	Median (IQR) Training Workouts before First Race in Year	Median (IQR) Days between First Workout and First Race in Year
2015	8 (5–10)	66 (42–99)	5 (2–8)	45 (22–78)	2 (1–4)	29 (14–53)
2016	8 (5–10)	67 (42–99)	5 (2–8)	48 (23–79)	2 (1–5)	32 (15–56)
2017	8 (5–11)	67 (43–100)	4 (2–8)	46 (22–77)	2 (1–4)	30 (15–54)
2018	8 (5–10)	68 (44–99)	5 (2–8)	45 (22–77)	2 (1–4)	30 (14–53)
2019	8 (5–10)	68 (45–101)	5 (2–8)	45 (22–78)	2 (0–4)	31 (15–55)
2020	8 (5–10)	69 (45–101)	4 (2–8)	48 (23–79)	2 (0–4)	30 (14–57)
2021	8 (6–10)	66 (42–99)	5 (2–8)	46 (22–77)	2 (1–5)	32 (16–55)
2022	8 (6–10)	67 (42–99)	5 (2–8)	45 (22–77)	2 (0–5)	33 (16–54)

**Table 3:** Median (interquartile range) days to first training workout, and to first race start, in each year from 2015 to 2022, for three age groups of horses.

	2-Year-Old Horses		3-Year-Old Horses		4-Year-Old-and-Older Horses	
Year	Median (IQR) Days to First Workout	Median (IQR) Days to First Race	Median (IQR) Days to First Workout	Median (IQR) Days to First Race	Median (IQR) Days to First Workout	Median (IQR) Days to First Race
2015	184 (138–245)	248 (200–298)	70 (15–133)	109 (31–171)	73 (18–124)	66 (17–139)
2016	182 (139–244)	247 (204–302)	64 (12–132)	107 (33–169)	67 (16–125)	70 (16–141)
2017	180 (138–243)	246 (202–299)	63 (15–130)	105 (30–168)	69 (19–125)	62 (18–139)
2018	181 (139–243)	244 (202–299)	62 (14–128)	109 (32–166)	67 (19–124)	56 (20–138)
2019	178 (137–240)	245 (202–297)	59 (12–125)	107 (27–167)	68 (17–124)	51 (15–138)
2020	192 (149–248)	255 (214–302)	54 (11–146)	87 (25–188)	66 (16–149)	40 (15–170)
2021	178 (136–240)	247 (204–298)	62 (10–130)	98 (27–171)	68 (15–126)	50 (16–140)
2022	165 (129–211)	245 (204–294)	52 (13–121)	92 (27–163)	67 (16–123)	48 (15–135)

## Data Availability

Data used belong to The Jockey Club and were licenced for use on a limited, non-transferable, and non-exclusive basis.

## References

[1] Allen S.E., Rosanowski S.M., Stirk A.J., Verheyen K.L.P. (2017). Description of veterinary events and risk factors for fatality in National Hunt flat racing Thoroughbreds in Great Britain (2000–2013). Equine Vet. J..

[2] Anthenill L.A., Stover S.M., Gardner I.A., Hill A.E. (2007). Risk factors for proximal sesamoid bone fractures associated with exercise history and horseshoe characteristics in Thoroughbred racehorses. Am. J. Vet. Res..

[3] Bailey C.J., Reid S.W., Hodgson D.R., Suann C.J., Rose R.J. (1997). Risk factors associated with musculoskeletal injuries in Australian thoroughbred racehorses. Prev. Vet. Med..

[4] Bailey C.J., Reid S.W., Hodgson D.R., Bourke J.M., Rose R.J. (1998). Flat, hurdle and steeple racing: Risk factors for musculoskeletal injury. Equine Vet. J..

[5] Boden L.A., Anderson G.A., Charles J.A., Morgan K.L., Morton J.M., Parkin T.D.H., Clarke A.F., Slocombe R.F. (2007). Risk factors for Thoroughbred racehorse fatality in flat starts in Victoria, Australia (1989–2004). Equine Vet. J..

[6] Boden L.A., Anderson G.A., Charles J.A., Morgan K.L., Morton J.M., Parkin T.D.H., Clarke A.F., Slocombe R.F. (2007). Risk factors for Thoroughbred racehorse fatality in jump starts in Victoria, Australia (1989–2004). Equine Vet. J..

[7] Bolwell C., Rogers C., Gee E., McIlwraith W. (2017). Epidemiology of Musculoskeletal Injury during Racing on New Zealand Racetracks 2005–2011. Animals.

[8] Cohen N.D., Berry S.M., Peloso J.G., Mundy G.D., Howard I.C. (2000). Association of high-speed exercise with racing injury in thoroughbreds. J. Am. Vet. Med. Assoc..

[9] Crawford K.L., Ahern B.J., Perkins N.R., Phillips C.J.C., Finnane A. (2020). The Effect of Combined Training and Racing High-Speed Exercise History on Musculoskeletal Injuries in Thoroughbred Racehorses: A Systematic Review and Meta-Analysis of the Current Literature. Animals.

[10] Cruz A.M., Poljak Z., Filejski C., Lowerison M.L., Goldie K., Martin S.W., Hurtig M.B. (2007). Epidemiologic characteristics of catastrophic musculoskeletal injuries in Thoroughbred racehorses. Am. J. Vet. Res..

[11] Georgopoulos S.P., Parkin T.D.H. (2016). Risk factors associated with fatal injuries in Thoroughbred racehorses competing in flat racing in the United States and Canada. J. Am. Vet. Med. Assoc..

[12] Hernandez J., Hawkins D.L., Scollay M.C. (2001). Race-start characteristics and risk of catastrophic musculoskeletal injury in Thoroughbred racehorses. J. Am. Vet. Med. Assoc..

[13] Hitchens P.L., Morrice-West A.V., Stevenson M.A., Whitton R.C. (2019). Meta-analysis of risk factors for racehorse catastrophic musculoskeletal injury in flat racing. Vet. J..

[14] Parkin T.D.H., Clegg P.D., French N.P., Proudman C.J., Riggs C.M., Singer E.R., Webbon P.M., Morgan K.L. (2006). Analysis of horse race videos to identify intra-race risk factors for fatal distal limb fracture. Prev. Vet. Med..

[15] Parkin T.D.H., Clegg P.D., French N.P., Proudman C.J., Riggs C.M., Singer E.R., Webbon P.M., Morgan K.L. (2004). Race- and course-level risk factors for fatal distal limb fracture in racing Thoroughbreds. Equine Vet. J..

[16] Parkin T.D.H., Clegg P.D., French N.P., Proudman C.J., Riggs C.M., Singer E.R., Webbon P.M., Morgan K.L. (2004). Horse-level risk factors for fatal distal limb fracture in racing Thoroughbreds in the UK. Equine Vet. J..

[17] Perkins N.R., Reid S.W.J., Morris R.S. (2005). Risk factors for musculoskeletal injuries of the lower limbs in Thoroughbred racehorses in New Zealand. N. Z. Vet. J..

[18] Rosanowski S.M., Chang Y.-M., Stirk A.J., Verheyen K.L.P. (2018). Risk factors for race-day fatality in flat racing Thoroughbreds in Great Britain (2000 to 2013). PLoS ONE.

[19] Zambruno T., Georgopoulos S.P., Boden L.A., Parkin T.D.H. (2020). Association between the administration of phenylbutazone prior to racing and musculoskeletal and fatal injuries in Thoroughbred racehorses in Argentina. J. Am. Vet. Med. Assoc..

[20] Lyle C.H., Uzal F.A., McGorum B.C., Aida H., Blissitt K.J., Case J.T., Charles J.T., Gardner I., Horadagoda N., Kusano K. (2011). Sudden death in racing Thoroughbred horses: An international multicentre study of post mortem findings. Equine Vet. J..

[21] Lyle C.H., Blissitt K.J., Kennedy R.N., Mc Gorum B.C., Newton J.R., Parkin T.D.H., Stirk A., Boden L.A. (2012). Risk factors for race-associated sudden death in Thoroughbred racehorses in the UK (2000–2007). Equine Vet. J..

[22] Boden L.A., Charles J.A., Slocombe R.F., Sandy J.R., Finnin P.J., Morton J.M., Clarke A.F. (2005). Sudden death in racing Thoroughbreds in Victoria, Australia. Equine Vet. J..

[23] Bennet E.D., Parkin T.D.H. (2022). Fifteen risk factors associated with sudden death in Thoroughbred racehorses in North America (2009–2021). J. Am. Vet. Med. Assoc..

[24] Brown C.M., Kaneene J.B., Taylor R.F. (1988). Sudden and unexpected death in horses and ponies: An analysis of 200 cases. Equine Vet. J..

[25] Verheyen K.L.R., Wood J.L.N. (2004). Descriptive epidemiology of fractures occurring in British Thoroughbred racehorses in training. Equine Vet. J..

[26] Nath L., Stent A., Elliott A., La Gerche A., Franklin S. (2022). Risk Factors for Exercise-Associated Sudden Cardiac Death in Thoroughbred Racehorses. Animals.

[27] Ayodele B.A., Hitchens P.L., Wong A.S.M., Mackie E.J., Whitton R.C. (2021). Microstructural properties of the proximal sesamoid bones of Thoroughbred racehorses in training. Equine Vet. J..

[28] Malekipour F., Hitchens P.L., Whitton R.C., Vee-Sin Lee P. (2022). Effects of in vivo fatigue-induced microdamage on local subchondral bone strains. J. Mech. Behav. Biomed. Mater..

[29] Morrice-West A.V., Hitchens P.L., Walmsley E.A., Tasker K., Lim S.L., Smith A.D., Whitton R.C. (2022). Relationship between Thoroughbred workloads in racing and the fatigue life of equine subchondral bone. Sci Rep.

[30] Morrice-West A.V., Hitchens P.L., Walmsley E.A., Wong A.S.M., Whitton R.C. (2021). Association of Thoroughbred Racehorse Workloads and Rest Practices with Trainer Success. Animals.

[31] Watts J. (2023). Equestrian Sport Should Be Brave and Proactive on Welfare to Maintain Public Acceptance.

[32] The Jockey Club (2023). The Jockey Club Releases Data from the Equine Injury Database for 2022.

